# Editorial Extracts and Gleanings

**Published:** 1874-01

**Authors:** A. R. Kilpatrick

**Affiliations:** Texas


By A. R. KILPATRICK, M.D., OF TEXAS.

*Treatment of Gastralgia.*—Dr. Soulin (France Medicale) strongly advocates for the treatment of gastralgia: 1. A poultice of ice for ten minutes, morning and evening, to the pit of the stomach; 2. A mustard plaster to the same spot immediately on removing the ice poultice, to be kept on as long as possible; 3. Pounded ice, to be taken morning and evening, a tablespoonful every five minutes for one hour; 4. A mustard bath with two pounds of mustard three times a week.

The ice of the poultice is to be chopped up in small bits and inclosed in an india-rubber bag. The ice taken internally must be pounded with sugar, or it may be prepared by an ice-man to the taste of the patient. It must be swallowed down suddenly, and not kept in the mouth, as it would lose its coldness. If a tablespoonful would be too much at a time, as we fancy it mostly would, several teaspoonfuls may be taken instead. The mustard bath Dr. Joulin regards as a powerful tonic.—*American Practitioner.*

*Atropia in Salivation.*—Dr. Wilhelm Ebstein (Breslau), concludes a paper on this subject in the Berlin Med. Wochenschrift June 23 as follows:

A work by my friend Paul Gurtzner in Pfluger's Archiv., furnishes the proof that irritation of the medula oblongata is followed by a marked increase of salivary secretion which is checked on the side in which the chorda tympani and sympathetic have been divided; on the other hand, it continues on the other side, where both or one of these nerves remain intact; so that the assumption of a salivary centre in the spinal cord is justifiable. * *

Of far greater practical importance is the therapeutic value of atropia in the treatment of salivation as experimental investigations have so clearly indicated. We have in atropia a means which markedly relieves the distress of salivation. When I give my patient a hypodermic injection of 0.0016 atropia, I assure him a perfect night's rest which would be else impossible on his constantly saturated pillow. In salivation, atropia is the proper narcotic.—*The Clinic.*

*Yellow Fever in New Orleans*, 1873.—Dr. Alfred W. Perry, Sanitary Inspector of New Orleans Board of Health, reports in the November number of the New Orleans Medical Journal, his success in checking the spread of yellow fever by carbolic acid, used freely and extensively as a disinfectant, in those parts ot the city where cases of the fever occurred.

The fever was introduced there on the 4th of July, by the mate of a Spanish vessel from Havana, which arrived on the 26th June, and the disease slowly spread. During the first week of Augnst, the Board of Health commenced extensive disinfection with carbolic acid, of all places where fever had been reported. The acid was sprinkled all over the squares infected, with hand sprinklers, using seventy gallons to the square. The streets were also sprinkled, using twenty gallons to every one hundred yards. This was repeated several times at intervals of five to ten days. Thirty squares and twenty-one half squares, where the fever had appeared, were gone over in this way, and in only seven of these areas did any subsequent cases occur. The same plan was pursucd in Mobile, with like results. The number of deaths from yellow fever in New Orleans was trifling, compared with former years, or with the smaller cities of Memphis, Shreveport, and towns in Texas.

*Puerperal Amaurosis—Its Importance as a Symptom.*—Dr. H. H. Williams, of Boston, gives two cases, in the Boston Medical and Surgical Journal, of this form of disease occurring in his own practice, and adds four others reported by Dr. F. Weber, of St.

Petersburg. Dr. Williams believes that puerperal amaurosis has some relation with puerperal convulsions, though he does not regard the blindness as invariably the forerunner of the convulsions. Eclampsia occurs more frequently than the amauroses, and often precedes it.

We regard the amaurosis as a very grave and important symptom, when it occurs before, or at the beginning of parturition, and we have observed that it attends nearly every case of eclampsia, during its progress. Frequently it supervenes several days before labor begins, and is accompanied, in some instances, with albuminuria. We have seen amaurosis in both primiparæ and multiparæ.

Dr. W. does not recommend any special treatment, only rest and the avoidance of whatever may induce cerebral congestion. He thinks an attack at one puerperal period does not involve the probability of its occurrence at a subsequent one. We have seen it occur two consecutive times in the same woman. We use the lancet to ward off an attack.

*Remarkable Mode of Infanticide.*—Professor J. S. Hughes, in the Irish Hospital Gazette, tells of cases of infanticide perpetrated by thrusting a sharp instrument, such as a strong needle, under the upper eyelid, so as to pierce the thin portion of the orbital plate of the frontal bone, and thereby reach the brain. This fatal thrust can be made without producing any noticeable mark or discoloration so as to lead to detection. The only symptom before death may be a convulsion, which, owing to its common occurrence, would not create suspicion. If a knowledge of this easy way of committing infanticide should become generally known, we may expect to hear of much loss of life thereby, and hereafter coroners should bear it in mind.

During the "Florida War" against the Seminoles, about 1836, a certain Lieutenant Lane, a brother of the notorious Jim Lane, of Kansas, killed himself in this way, by thrusting the point of his sword into his brain at the inner canthus.

*Chloral in Asthma.*—Dr. Theo. Williams, of London, details cases of spasmodic asthma which he relieved promptly with chloral, after other remedies had failed, and after protracted suffering.

*A Vacancy for a Doctor.*—The position of medical adviser to the tribe of Tulare Indians, in California, is vacant. The late incumbent had intrusted to his care a number of sick Indians, all of whom unfortunately died; upon which, a grand council was held, and the medicine man was condemned to death and thereupon promptly executed.

*A Post-obit.*—A convicted murderer under sentence of death, in Georgia, has sold his body to a medical gentleman for ten dollars, which he has expended for toilet articles.

*Quinine During Pregnancy.*—Much discussion has been going on as to whether quinine is a uterine excitant or oxytocic, and can be safely used during pregnancy. Monseur Monteverdi advises to use opium in such patients with the quinine, which is very proper. Our impression is, after noticing it for a long time, that the medicine is used only in sickly people, living in malarious districts, with broken-down systems; and in cases where abortions and premature labors come on, they are owing to the unhealthy, anemic, debilitated mother, and the abortion is a *post hoc*, but not a *propter hoc*, of the medicine.

*Belladonna Plaster in Obstinate Vomiting and Sea-Sickness.*—Dr. Guéneau de Mussy recommends in obstinate vomiting and sea-sickness a plaster made of diachylon and theriac plaster, each two parts, and extract of belladonna one part, well mixed and spread on cloth or sheepskin four inches square, and applied to the epigastrium, and may be worn from twelve to fifteen days without being renewed. Several cases illustrative of its beneficial effects are detailed by Dr. G., both in vomiting from gastric derangement, or pregnancy, and also in persons great annoyed previously by sea-sickness.

*For Cholera.*—The following formula is used in Dacca and other places in the East Indies, as a remedy for cholera in all stages: R. Argenti nitras, opii pulv., camphoræ, aa gr. i.; terebinth. chia, (Mastich) q. s. M. ft. pil. No. ii. Give one every have hour till relieved.

